# Supplementary material for: Associations of the Short Physical Performance Battery (SPPB) with Adverse Health Outcomes in Older Adults: A 14-Year Follow-Up from the English Longitudinal Study of Ageing (ELSA)
Source: Int J Environ Res Public Health. 2022 Dec 6;19(23):16319. doi: 10.3390/ijerph192316319 (PMC9739256; doi:10.3390/ijerph192316319)
Supplement: Supplementary file 1 [file ijerph-19-16319-s001.zip › ijerph-2055273-supplementary.pdf]

Supplementary Materials

Table S1. STROBE checklist.

STROBE Statement—checklist of items that should be included in reports of observational studies

	Item No	Recommendation
**Title and abstract**	1	(*a*) Indicate the study's design with a commonly used term in the title or the abstract **(page 1)**
		(*b*) Provide in the abstract an informative and balanced summary of what was done and what was found **(page 1)**
**Introduction**		
Background/rationale	2	Explain the scientific background and rationale for the investigation being reported **(pages 1–2)**
Objectives	3	State specific objectives, including any prespecified hypotheses **(page 2)**
**Methods**		
Study design	4	Present key elements of study design early in the paper **(page 2)**
Setting	5	Describe the setting, locations, and relevant dates, including periods of recruitment, exposure, follow-up, and data collection **(page 2)**
Participants	6	(*a*) *Cohort study*—Give the eligibility criteria, and the sources and methods of selection of participants. Describe methods of follow-up **(page 2)**<br>*Case-control study*—Give the eligibility criteria, and the sources and methods of case ascertainment and control selection. Give the rationale for the choice of cases and controls<br>*Cross-sectional study*—Give the eligibility criteria, and the sources and methods of selection of participants
		(*b*) *Cohort study*—For matched studies, give matching criteria and number of exposed and unexposed **N/A**<br>*Case-control study*—For matched studies, give matching criteria and the number of controls per case
Variables	7	Clearly define all outcomes, exposures, predictors, potential confounders, and effect modifiers. Give diagnostic criteria, if applicable **(pages 2–3 and Supplementary Materials pages 4–6)**
Data sources/ measurement	8*	For each variable of interest, give sources of data and details of methods of assessment (measurement). Describe comparability of assessment methods if there is more than one group **(pages 2–3 and Supplementary Materials pages 4–6)**
Bias	9	Describe any efforts to address potential sources of bias **(pages 3–4)**
Study size	10	Explain how the study size was arrived at **(pages 2–4 and Supplementary Materials page 12)**
Quantitative variables	11	Explain how quantitative variables were handled in the analyses. If applicable, describe which groupings were chosen and why **(pages 2–4)**
Statistical methods	12	(*a*) Describe all statistical methods, including those used to control for confounding **(pages 3–4 and Supplementary Materials pages 7–8)**

	(*b*) Describe any methods used to examine subgroups and interactions **(pages 3–4)**
	(*c*) Explain how missing data were addressed **(page 4)**
	(*d*) *Cohort study*—If applicable, explain how loss to follow-up was addressed **(pages 3–4)**<br>*Case-control study*—If applicable, explain how matching of cases and controls was addressed<br>*Cross-sectional study*—If applicable, describe analytical methods taking account of sampling strategy
	(*e*) Describe any sensitivity analyses **(page 4)**

Continued on next page

**Results**		
Participants	13*	(a) Report numbers of individuals at each stage of study—eg numbers potentially eligible, examined for eligibility, confirmed eligible, included in the study, completing follow-up, and analysed **(Supplementary Materials page 12)**
		(b) Give reasons for non-participation at each stage **(Supplementary Materials page 12)**
		(c) Consider use of a flow diagram **(Supplementary Materials page 12)**
Descriptive data	14*	(a) Give characteristics of study participants (eg demographic, clinical, social) and information on exposures and potential confounders **(pages 4–6)**
		(b) Indicate number of participants with missing data for each variable of interest **(Supplementary Materials pages 9–11 and 14–16)**
		(c) *Cohort study*—Summarise follow-up time (eg, average and total amount) **(page 2 and Supplementary Materials page 13)**
Outcome data	15*	*Cohort study*—Report numbers of outcome events or summary measures over time **(pages 4–6 and Supplementary Materials page 13)**
		Case-control study—Report numbers in each exposure category, or summary measures of exposure
		Cross-sectional study—Report numbers of outcome events or summary measures
Main results	16	(*a*) Give unadjusted estimates and, if applicable, confounder-adjusted estimates and their precision (eg, 95% confidence interval). Make clear which confounders were adjusted for and why they were included **(pages 6–11 and Supplementary Materials pages 17–29)**
		(*b*) Report category boundaries when continuous variables were categorized **(pages 2–4)**
		(*c*) If relevant, consider translating estimates of relative risk into absolute risk for a meaningful time period **N/A**
Other analyses	17	Report other analyses done—eg analyses of subgroups and interactions, and sensitivity analyses **(pages 6–11 and Supplementary Materials pages 17–29)**
**Discussion**		
Key results	18	Summarise key results with reference to study objectives **(page 12)**
Limitations	19	Discuss limitations of the study, taking into account sources of potential bias or imprecision. Discuss both direction and magnitude of any potential bias **(page 13)**
Interpretation	20	Give a cautious overall interpretation of results considering objectives, limitations, multiplicity of analyses, results from similar studies, and other relevant evidence **(pages 12–13)**
Generalisability	21	Discuss the generalisability (external validity) of the study results **(pages 12–13)**
**Other information**		
Funding	22	Give the source of funding and the role of the funders for the present study and, if applicable, for the original study on which the present article is based **(page 14)**

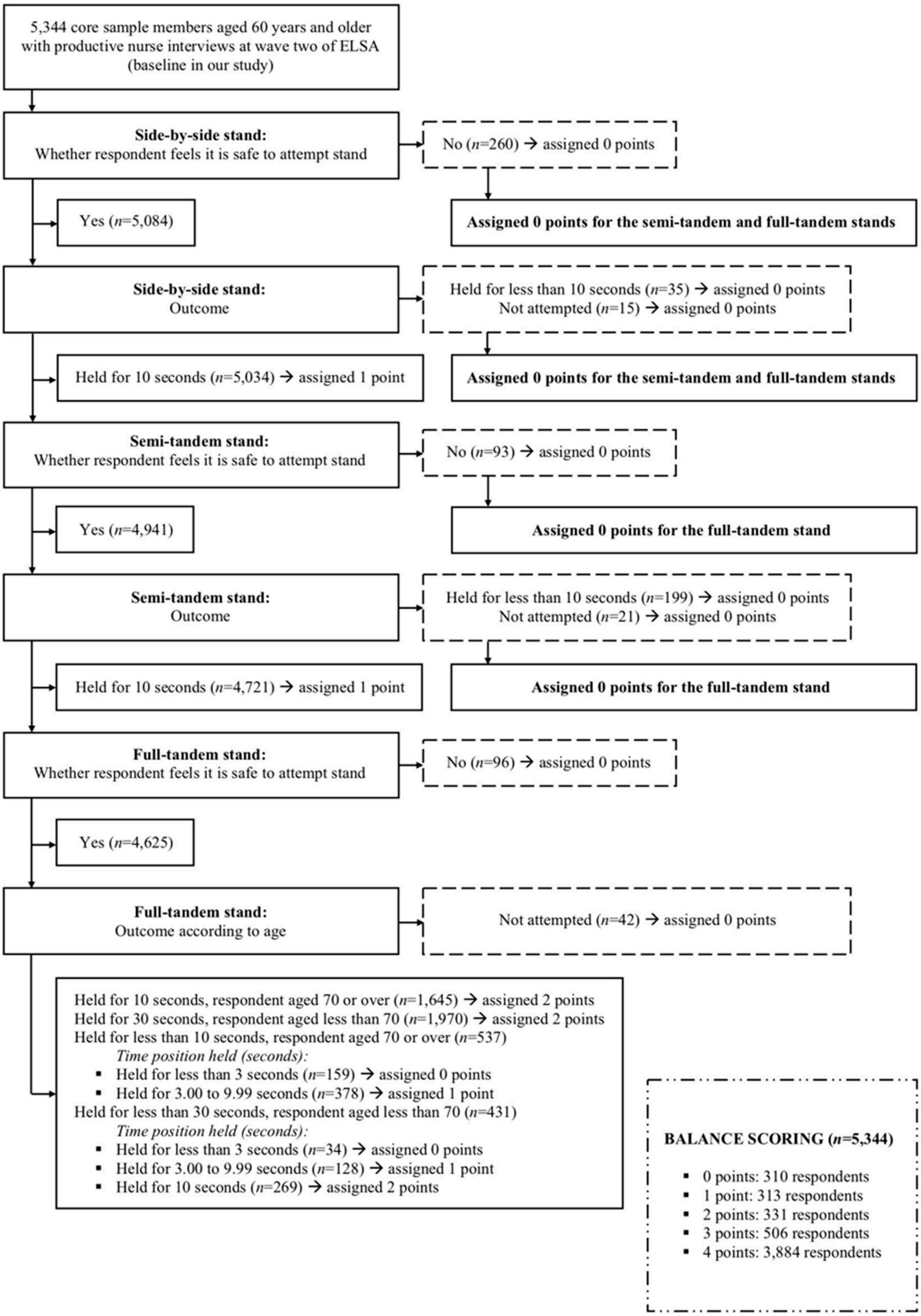

Figure S1. Flowchart summarising the balance test scoring system.

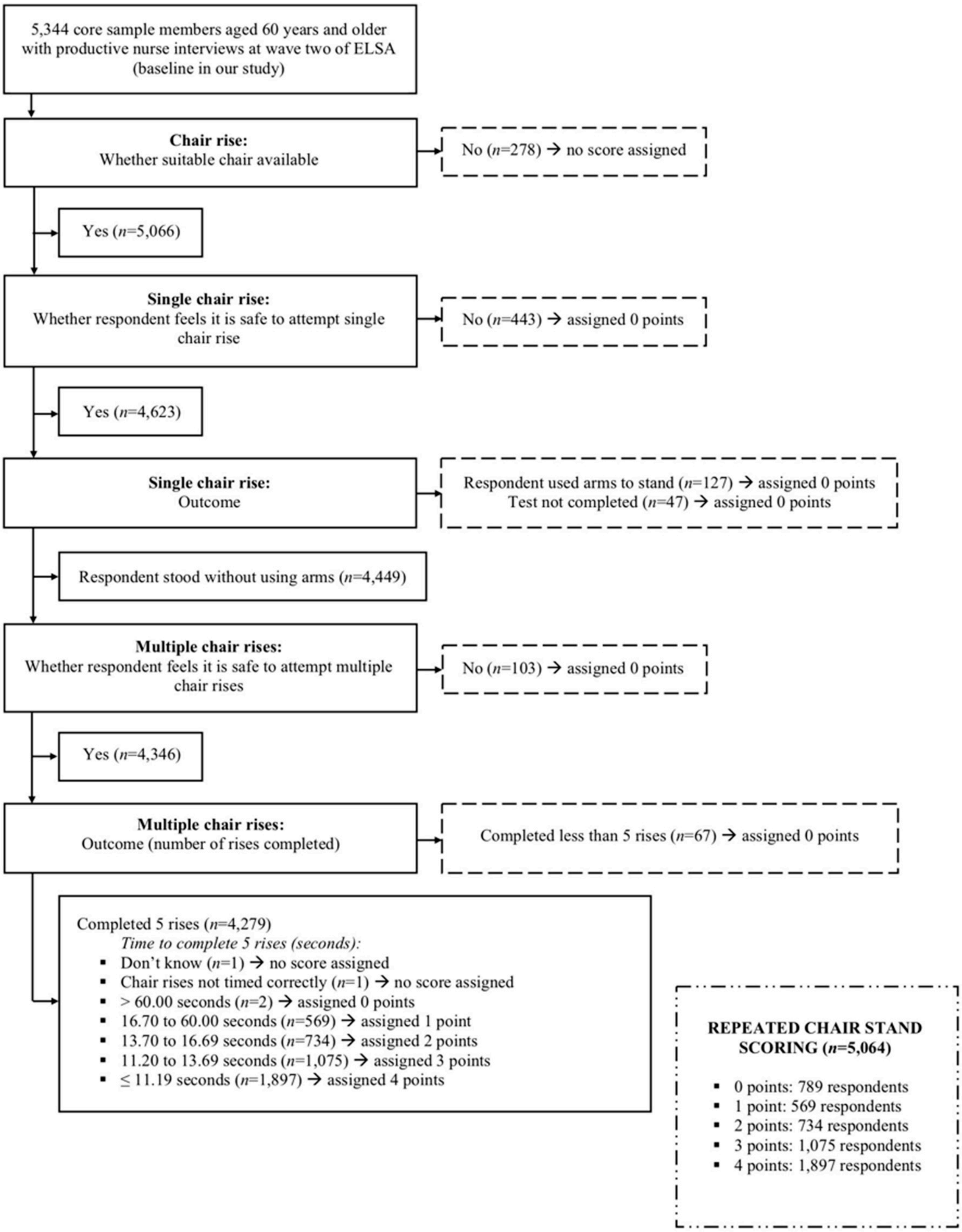

Figure S2. Flowchart summarising the repeated chair stand test scoring system.

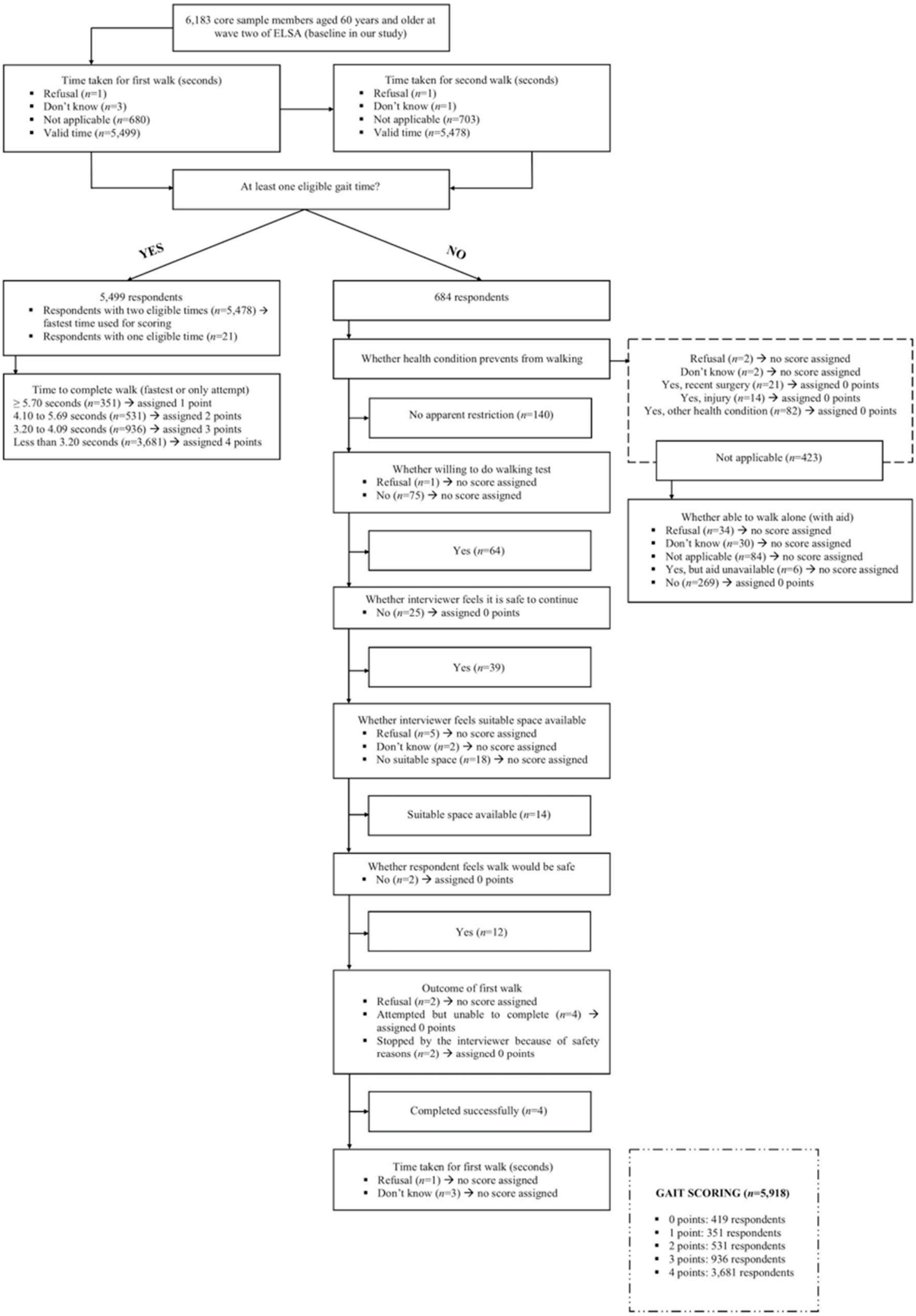

Figure S3. Flowchart summarising the gait test scoring system.

Table S2. Specification for multilevel models with total SPPB score as the exposure.

Model	Specification
Model 1	Univariable: total SPPB score (exposure) and outcome only
Model 2	Model 1 plus continuous linear time variable
Model 3	Model 2 plus continuous quadratic time variable [1]
Model 4	Model 3 plus socio-demographic covariates (age, biological sex, ethnicity, marital status, employment status, education, and wealth)
Model 5	Model 4 plus health-related covariates (physical activity, BMI, cognitive function, and depressive symptoms)
Model 6	Model 5 plus outcome from wave two
Model 7	Model 6 plus interaction between total SPPB score and age
Model 8	Model 6 plus interaction between total SPPB score and biological sex
Model 9	Model 6 plus interaction between total SPPB score and time
Model 10	Model 9 plus interaction between total SPPB score and quadratic time
Model 11 [2]	Model 6 plus significant interactions in Model 7–10

BMI, body-mass index; *SPPB*, Short Physical Performance Battery.

[1] The quadratic time variable was subsequently excluded from models if there was no evidence of non-linearity.

[2] Only performed if there was more than one significant interaction in Model 7–10.

Table S3. Specification for multilevel models with SPPB domain scores (balance, repeated chair stands, and gait) as the exposures.

Model	Specification
Model 1	Univariable: exposure(s) [1] and outcome only
Model 2	Model 1 plus continuous linear time variable
Model 3	Model 2 plus continuous quadratic time variable [2]
Model 4	Model 3 plus socio-demographic covariates (age, biological sex, ethnicity, marital status, employment status, education, and wealth)
Model 5	Model 4 plus health-related covariates (physical activity, BMI, cognitive function, and depressive symptoms)
Model 6	Model 5 plus outcome from wave two

BMI, body-mass index; *SPPB*, Short Physical Performance Battery.

[1] Balance score, repeated chair stand score, and gait score. Models were performed i) separately for each exposure; and ii) with all exposures entered simultaneously.

[2] The quadratic time variable was subsequently excluded from models if there was no evidence of non-linearity.

Table S4. Patterns of missing data prior to multiple imputation.

Variable	Missing	Total	Percent Missing
Total SPPB score	0	34,524	0.00
Balance score	0	34,524	0.00
Repeated Chair Stand score	0	34,524	0.00
Gait score	0	34,524	0.00
Age	0	34,524	0.00
Biological sex	0	34,524	0.00
Living status	0	34,524	0.00
Total SPPB score × Age	0	34,524	0.00
Total SPPB score × Biological sex	0	34,524	0.00
Ethnicity	7	34,524	0.02
Marital status	7	34,524	0.02
Employment status	77	34,524	0.22
Education	3,381	34,524	9.79
Wealth	357	34,524	1.03
Physical activity	7	34,524	0.02
Body-mass index	2,128	34,524	6.16
Time orientation	70	34,524	0.20
Immediate and delayed recall	98	34,524	0.28
Prospective memory	28	34,524	0.08
Verbal fluency	56	34,524	0.16
Processing speed	1,071	34,524	3.10
Processing efficiency	1,071	34,524	3.10
Depressive symptoms	371	34,524	1.07
Mobility impairments (follow-up)	14,121	34,524	40.90
ADL disabilities (follow-up)	14,116	34,524	40.89
IADL disabilities (follow-up)	14,116	34,524	40.89
Falls (follow-up)	14,866	34,524	43.06
Mobility impairments (baseline)	7	34,524	0.02
ADL disabilities (baseline)	7	34,524	0.02
IADL disabilities (baseline)	7	34,524	0.02
Falls (baseline)	21	34,524	0.06
Self-reported general health	7	34,524	0.02
Limiting long-standing illness	35	34,524	0.10
Occupational class	630	34,524	1.82
Alcohol consumption	3,794	34,524	10.99
Smoking status	28	34,524	0.08
Height	1,687	34,524	4.89
Weight	1,323	34,524	3.83

SPPB, Short Physical Performance Battery; *ADL*, Activities of Daily Living; *IADL*, Instrumental Activities of Daily Living.

Table S5. Number of observations imputed per dataset.

Variable	Complete	Incomplete	Imputed	Total
Total SPPB score	4,932	0	0	4,932
Balance score	4,932	0	0	4,932
Repeated Chair Stand score	4,932	0	0	4,932
Gait score	4,932	0	0	4,932
Age	4,932	0	0	4,932
Biological sex	4,932	0	0	4,932
Living status	4,932	0	0	4,932
Total SPPB score × Age	4,932	0	0	4,932
Total SPPB score × Biological sex	4,932	0	0	4,932
Ethnicity	4,931	1	1	4,932
Marital status	4,931	1	1	4,932
Employment status	4,921	11	11	4,932
Education	4,449	483	483	4,932
Wealth	4,881	51	51	4,932
Physical activity	4,931	1	1	4,932
Body-mass index	4,628	304	304	4,932
Time orientation	4,922	10	10	4,932
Immediate and delayed recall	4,918	14	14	4,932
Prospective memory	4,928	4	4	4,932
Verbal fluency	4,924	8	8	4,932
Processing speed	4,779	153	153	4,932
Processing efficiency	4,779	153	153	4,932
Depressive symptoms	4,879	53	53	4,932
Mobility impairments (wave three)	4,179	753	753	4,932
Mobility impairments (wave four)	3,610	1,322	1,322	4,932
Mobility impairments (wave five)	3,295	1,637	1,637	4,932
Mobility impairments (wave six)	2,950	1,982	1,982	4,932
Mobility impairments (wave seven)	2,515	2,417	2,417	4,932
Mobility impairments (wave eight)	2,111	2,821	2,821	4,932
Mobility impairments (wave nine)	1,743	3,189	3,189	4,932
ADL disabilities (wave three)	4,181	751	751	4,932
ADL disabilities (wave four)	3,610	1,322	1,322	4,932
ADL disabilities (wave five)	3,296	1,636	1,636	4,932
ADL disabilities (wave six)	2,950	1,982	1,982	4,932
ADL disabilities (wave seven)	2,515	2,417	2,417	4,932
ADL disabilities (wave eight)	2,113	2,819	2,819	4,932
ADL disabilities (wave nine)	1,743	3,189	3,189	4,932
IADL disabilities (wave three)	4,181	751	751	4,932
IADL disabilities (wave four)	3,610	1,322	1,322	4,932
IADL disabilities (wave five)	3,296	1,636	1,636	4,932
IADL disabilities (wave six)	2,950	1,982	1,982	4,932
IADL disabilities (wave seven)	2,515	2,417	2,417	4,932
IADL disabilities (wave eight)	2,113	2,819	2,819	4,932
IADL disabilities (wave nine)	1,743	3,189	3,189	4,932
Falls (wave three)	4,123	809	809	4,932
Falls (wave four)	3,510	1,422	1,422	4,932
Falls (wave five)	3,164	1,768	1,768	4,932
Falls (wave six)	2,817	2,115	2,115	4,932
Falls (wave seven)	2,390	2,542	2,542	4,932

Falls (wave eight)	2,006	2,926	2,926	4,932
Falls (wave nine)	1,648	3,284	3,284	4,932
Mobility impairments (baseline)	4,931	1	1	4,932
ADL disabilities (baseline)	4,931	1	1	4,932
IADL disabilities (baseline)	4,931	1	1	4,932
Falls (baseline)	4,929	3	3	4,932
Self-reported general health	4,931	1	1	4,932
Limiting long-standing illness	4,927	5	5	4,932
Occupational class	4,842	90	90	4,932
Alcohol consumption	4,390	542	542	4,932
Smoking status	4,928	4	4	4,932
Height	4,691	241	241	4,932
Weight	4,743	189	189	4,932

SPPB, Short Physical Performance Battery; *ADL*, Activities of Daily Living; *IADL*, Instrumental Activities of Daily Living.
Note: The gait score was omitted because of collinearity.

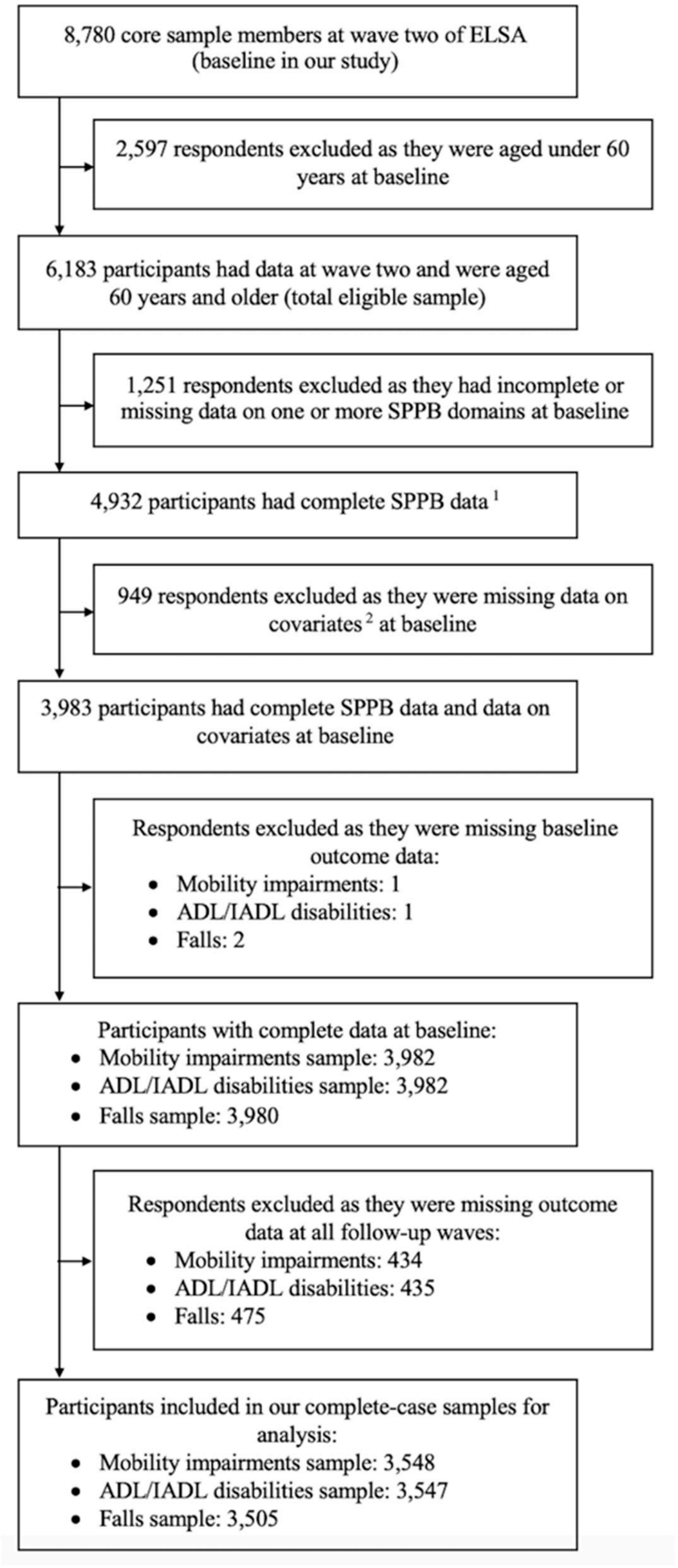

Figure S4. Flow of study members into the complete-case analytical samples.

ELSA, English Longitudinal Study of Ageing; *SPPB*, Short Physical Performance Battery; *ADL*, Activities of Daily Living; *IADL*, Instrumental Activities of Daily Living.

[1] This was the sample size used for multiple imputation to replace missing data on covariates (treated as time-invariant) and outcomes at all follow-up waves; exposure data were not imputed.

[2] Covariates were age, biological sex, ethnicity, marital status, employment status, education, wealth, physical activity, body-mass index, cognitive function, and depressive symptoms.

Table S6. Descriptive statistics for mobility impairments, ADL disabilities, IADL disabilities, and falls at each wave of follow-up.

	**Overall sample**
Mobility impairments (*n*=3,548; 16,934 observations), mean (SD)	
Wave three (*n*=3,401)	2.1 (2.5)
Wave four (*n*=2,978)	2.2 (2.5)
Wave five (*n*=2,726)	2.4 (2.7)
Wave six (*n*=2,459)	2.6 (2.8)
Wave seven (*n*=2,117)	2.6 (2.8)
Wave eight (*n*=1,778)	2.6 (2.8)
Wave nine (*n*=1,475)	2.8 (2.9)
ADL disabilities (*n*=3,547; 16,934 observations), mean (SD)	
Wave three (*n*=3,401)	0.4 (0.9)
Wave four (*n*=2,977)	0.4 (1.0)
Wave five (*n*=2,727)	0.5 (1.1)
Wave six (*n*=2,459)	0.5 (1.1)
Wave seven (*n*=2,117)	0.5 (1.2)
Wave eight (*n*=1,778)	0.5 (1.2)
Wave nine (*n*=1,475)	0.6 (1.3)
IADL disabilities (*n*=3,547; 16,934 observations), mean (SD)	
Wave three (*n*=3,401)	0.4 (1.0)
Wave four (*n*=2,977)	0.5 (1.1)
Wave five (*n*=2,727)	0.6 (1.3)
Wave six (*n*=2,459)	0.7 (1.4)
Wave seven (*n*=2,117)	0.7 (1.4)
Wave eight (*n*=1,778)	0.8 (1.6)
Wave nine (*n*=1,475)	0.9 (1.7)
Falls (*n*=3,505; 16,332 observations), *n* (% Yes)	
Wave three (*n*=3,364)	976 (29.0)
Wave four (*n*=2,898)	780 (26.9)
Wave five (*n*=2,625)	782 (29.8)
Wave six (*n*=2,351)	722 (30.7)
Wave seven (*n*=2,016)	634 (31.4)
Wave eight (*n*=1,688)	561 (33.2)
Wave nine (*n*=1,390)	476 (34.3)

SD, standard deviation; *n*, number of participants; *ADL*, Activities of Daily Living; *IADL*, Instrumental Activities of Daily Living.

Note: All values are weighted estimates. There are different numbers of participants with complete data on all variables at each wave as the number of individuals with available outcome data varied by timepoint.

Table S7. Baseline characteristics of the complete-case samples used for analyses versus the samples excluded due to missing data (unweighted).

	Mobility impairments			ADL/IADL disabilities			Falls		
	**Included (*n*=3,548)**	**Excluded (*n*=2,635)**	***p* value**	**Included (*n*=3,547)**	**Excluded (*n*=2,636)**	***p* value**	**Included (*n*=3,505)**	**Excluded (*n*=2,678)**	***p* value**
Age, mean (SD) [1]	70.3 (7.4)	72.8 (8.4)	<0.001	70.3 (7.4)	72.8 (8.4)	<0.001	70.3 (7.4)	72.9 (8.4)	<0.001
Observations, *n*	3,548	2,635		3,547	2,636		3,505	2,678	
Biological sex, *n* (%)			<0.001			<0.001			<0.001
Male	1,646 (46.4)	1,105 (41.9)		1,646 (46.4)	1,105 (41.9)		1,628 (46.4)	1,123 (41.9)	
Female	1,902 (53.6)	1,530 (58.1)		1,901 (53.6)	1,531 (58.1)		1,877 (53.6)	1,555 (58.1)	
Observations, *n*	3,548	2,635		3,547	2,636		3,505	2,678	
Ethnicity, *n* (%)			<0.001			<0.001			<0.001
White	3,502 (98.7)	2,548 (96.8)		3,501 (98.7)	2,549 (96.8)		3,460 (98.7)	2,590 (96.8)	
non-White	46 (1.3)	85 (3.2)		46 (1.3)	85 (3.2)		45 (1.3)	86 (3.2)	
Observations, *n*	3,548	2,633		3,547	2,634		3,505	2,676	
Marital status, *n* (%)			<0.001			<0.001			<0.001
Single, that is never married	159 (4.5)	138 (5.2)		159 (4.5)	138 (5.2)		157 (4.5)	140 (5.2)	
Legally separated	31 (0.9)	21 (0.8)		31 (0.9)	21 (0.8)		31 (0.9)	21 (0.8)	
Divorced	256 (7.2)	204 (7.7)		256 (7.2)	204 (7.7)		256 (7.3)	204 (7.6)	
Widowed	803 (22.6)	735 (27.9)		803 (22.6)	735 (27.9)		792 (22.6)	746 (27.9)	
Married, first and only marriage	1,969 (55.5)	1,301 (49.4)		1,968 (55.5)	1,302 (49.4)		1,944 (55.5)	1,326 (49.5)	
Remarried, second or later marriage	330 (9.3)	235 (8.9)		330 (9.3)	235 (8.9)		325 (9.3)	240 (9.0)	
Observations, *n*	3,548	2,634		3,547	2,635		3,505	2,677	
Employment, *n* (%)			<0.001			<0.001			<0.001
Retired	2,599 (73.3)	1,895 (72.3)		2,598 (73.2)	1,896 (72.3)		2,568 (73.3)	1,926 (72.3)	
Unemployed	10 (0.3)	9 (0.3)		10 (0.3)	9 (0.3)		10 (0.3)	9 (0.3)	
Permanently sick or disabled	91 (2.6)	157 (6.0)		91 (2.6)	157 (6.0)		90 (2.6)	158 (5.9)	
Looking after home or family	344 (9.7)	295 (11.3)		344 (9.7)	295 (11.3)		337 (9.6)	302 (11.3)	
Semi-retired	23 (0.6)	15 (0.6)		23 (0.6)	15 (0.6)		23 (0.7)	15 (0.6)	
Employed	358 (10.1)	197 (7.5)		358 (10.1)	197 (7.5)		357 (10.2)	198 (7.4)	
Self-employed	123 (3.5)	53 (2.0)		123 (3.5)	53 (2.0)		120 (3.4)	56 (2.1)	
Observations, *n*	3,548	2,621		3,547	2,622		3,505	2,664	
Education, *n* (%)			<0.001			<0.001			<0.001

No qualification	1,589 (44.8)	1,211 (59.4)		1,588 (44.8)	1,212 (59.4)		1,564 (44.6)	1,236 (59.4)	
Secondary or lower	1,036 (29.2)	472 (23.2)		1,036 (29.2)	472 (23.1)		1,026 (29.3)	482 (23.2)	
Higher education below degree	485 (13.7)	166 (8.1)		485 (13.7)	166 (8.1)		480 (13.7)	171 (8.2)	
Degree or equivalent	438 (12.3)	189 (9.3)		438 (12.3)	189 (9.3)		435 (12.4)	192 (9.2)	
Observations, *n*	3,548	2,038		3,547	2,039		3,505	2,081	
Wealth, *n* (%)			<0.001			<0.001			<0.001
1st quintile (lowest)	551 (15.5)	659 (25.6)		551 (15.5)	659 (25.6)		544 (15.5)	666 (25.4)	
2nd quintile	663 (18.7)	571 (22.2)		663 (18.7)	571 (22.2)		660 (18.8)	574 (21.9)	
3rd quintile	712 (20.1)	517 (20.1)		712 (20.1)	517 (20.1)		702 (20.0)	527 (20.1)	
4th quintile	784 (22.1)	427 (16.6)		784 (22.1)	427 (16.6)		771 (22.0)	440 (16.8)	
5th quintile (highest)	838 (23.6)	400 (15.5)		837 (23.6)	401 (15.6)		828 (23.6)	410 (15.7)	
Observations, *n*	3,548	2,574		3,547	2,575		3,505	2,617	
Physical activity, *n* (%)			<0.001			<0.001			<0.001
Inactive	221 (6.2)	431 (16.9)		220 (6.2)	432 (16.9)		211 (6.0)	441 (17.0)	
Mild activity	528 (14.9)	545 (21.3)		528 (14.9)	545 (21.3)		521 (14.9)	552 (21.3)	
Moderate activity	1,816 (51.2)	1,127 (44.1)		1,816 (51.2)	1,127 (44.1)		1,796 (51.2)	1,147 (44.2)	
Vigorous activity	983 (27.7)	450 (17.6)		983 (27.7)	450 (17.6)		977 (27.9)	456 (17.6)	
Observations, *n*	3,548	2,553		3,547	2,554		3,505	2,596	
BMI (kg/m^2), mean (SD)	27.9 (4.7)	27.6 (4.8)	0.037	27.9 (4.7)	27.6 (4.8)	0.034	27.9 (4.7)	27.5 (4.8)	0.008
Observations, *n*	3,548	1,436		3,547	1,437		3,505	1,479	
Cognitive function, mean (SD)	0.1 (1.0)	-0.2 (1.0)	<0.001	0.1 (1.0)	-0.2 (1.0)	<0.001	0.1 (0.9)	-0.2 (1.0)	<0.001
Observations, *n*	3,548	2,285		3,547	2,286		3,505	2,328	
Depressive symptoms (range 0-8), mean (SD)	1.4 (1.8)	1.9 (2.1)	<0.001	1.4 (1.8)	1.9 (2.1)	<0.001	1.4 (1.8)	1.9 (2.1)	<0.001
Observations, *n*	3,548	2,451		3,547	2,452		3,505	2,494	
Total SPPB score (range 0-12), mean (SD)	9.8 (2.7)	8.0 (3.8)	<0.001	9.8 (2.7)	8.0 (3.8)	<0.001	9.8 (2.6)	8.0 (3.8)	<0.001
Observations, *n*	3,548	1,384		3,547	1,385		3,505	1,427	
Balance score, *n* (%)			<0.001			<0.001			<0.001
0	67 (1.9)	243 (13.5)		67 (1.9)	243 (13.5)		66 (1.9)	244 (13.3)	
1	160 (4.5)	153 (8.5)		160 (4.5)	153 (8.5)		154 (4.4)	159 (8.6)	
2	193 (5.4)	138 (7.7)		193 (5.4)	138 (7.7)		187 (5.3)	144 (7.8)	
3	316 (8.9)	190 (10.6)		316 (8.9)	190 (10.6)		312 (8.9)	194 (10.5)	

4	2,812 (79.3)	1,072 (59.7)		2,811 (79.3)	1,073 (59.7)		2,786 (79.5)	1,098 (59.7)	
Observations, *n*	3,548	1,796		3,547	1,797		3,505	1,839	
Repeated Chair Stand score, *n* (%)			<0.001			<0.001			<0.001
0	385 (10.9)	404 (26.6)		385 (10.9)	404 (26.6)		376 (10.7)	413 (26.5)	
1	372 (10.5)	197 (13.0)		372 (10.5)	197 (13.0)		364 (10.4)	205 (13.1)	
2	535 (15.1)	199 (13.1)		535 (15.1)	199 (13.1)		526 (15.0)	208 (13.3)	
3	798 (22.5)	277 (18.3)		797 (22.5)	278 (18.3)		794 (22.7)	281 (18.0)	
4	1,458 (41.1)	439 (29.0)		1,458 (41.1)	439 (28.9)		1,445 (41.2)	452 (29.0)	
Observations, *n*	3,548	1,516		3,547	1,517		3,505	1,559	
Gait score, *n* (%)			<0.001			<0.001			<0.001
0	124 (3.5)	295 (12.4)		124 (3.5)	295 (12.4)		121 (3.5)	298 (12.3)	
1	129 (3.6)	222 (9.4)		129 (3.6)	222 (9.4)		124 (3.5)	227 (9.4)	
2	273 (7.7)	258 (10.9)		273 (7.7)	258 (10.9)		268 (7.6)	263 (10.9)	
3	554 (15.6)	382 (16.1)		553 (15.6)	383 (16.2)		544 (15.5)	392 (16.2)	
4	2,468 (69.6)	1,213 (51.2)		2,468 (69.6)	1,213 (51.2)		2,448 (69.8)	1,233 (51.1)	
Observations, *n*	3,548	2,370		3,547	2,371		3,505	2,413	
Mobility impairments (range 0-10), mean (SD)	2.0 (2.4)	3.0 (2.9)	<0.001	–	–		–	–	
Observations, *n*	3,548	2,633		–	–		–	–	
ADL disabilities (range 0-6), mean (SD)	–	–		0.3 (0.8)	0.7 (1.3)	<0.001	–	–	
Observations, *n*	–	–		3,547	2,634		–	–	
IADL disabilities (range 0-7), mean (SD)	–	–		0.3 (0.8)	0.9 (1.5)	<0.001	–	–	
Observations, *n*	–	–		3,547	2,634		–	–	
Falls, *n* (%)									<0.001
No	–	–		–	–		2,439 (69.6)	1,651 (63.7)	
Yes	–	–		–	–		1,066 (30.4)	942 (36.3)	
Observations, *n*	–	–		–	–		3,505	2,593	

n, number of participants; *SD*, standard deviation; *BMI*, body-mass index; *SPPB*, Short Physical Performance Battery; *ADL*, Activities of Daily Living; *IADL*, Instrumental Activities of Daily Living.

[1] Age was collapsed to 90 for participants aged 90+ years.

Note: The samples with complete data and those with missing data for each outcome add up to the total eligible sample at wave two (*n*=6,183; see Figure S4).

Table S8. Odds ratios for mobility impairments, ADL disabilities, and IADL disabilities per one-point increase in total SPPB score, using repeated measures outcomes from seven waves of follow-up.

	**OR (95% CI)**	***p* value**
Mobility impairments (*n*=3,548; 16,934 observations)		
Model 1: Univariable association	0.51 (0.48, 0.54)	<0.001
Model 2: Model 1 plus continuous linear time variable	0.49 (0.46, 0.52)	<0.001
Model 3: Model 2 plus continuous quadratic time variable [1]	0.49 (0.46, 0.52)	<0.001
Model 4: Model 2 plus socio-demographic covariates [2]	0.59 (0.55, 0.62)	<0.001
Model 5: Model 4 plus health-related covariates [3]	0.69 (0.65, 0.73)	<0.001
Model 6: Model 5 plus mobility impairments at wave two	0.79 (0.75, 0.83)	<0.001
ADL disabilities (*n*=3,547; 16,934 observations)		
Model 1: Univariable association	0.60 (0.58, 0.62)	<0.001
Model 2: Model 1 plus continuous linear time variable	0.57 (0.55, 0.59)	<0.001
Model 3: Model 2 plus continuous quadratic time variable [1]	0.57 (0.55, 0.59)	<0.001
Model 4: Model 2 plus socio-demographic covariates [2]	0.63 (0.61, 0.66)	<0.001
Model 5: Model 4 plus health-related covariates [3]	0.73 (0.70, 0.76)	<0.001
Model 6: Model 5 plus ADL disabilities at wave two	0.81 (0.78, 0.85)	<0.001
IADL disabilities (*n*=3,547; 16,934 observations)		
Model 1: Univariable association	0.59 (0.56, 0.61)	<0.001
Model 2: Model 1 plus continuous linear time variable	0.53 (0.51, 0.56)	<0.001
Model 3: Model 2 plus continuous quadratic time variable [1]	0.53 (0.51, 0.56)	<0.001
Model 4: Model 2 plus socio-demographic covariates [2]	0.64 (0.61, 0.66)	<0.001
Model 5: Model 4 plus health-related covariates [3]	0.73 (0.70, 0.76)	<0.001
Model 6: Model 5 plus IADL disabilities at wave two	0.81 (0.78, 0.84)	<0.001

ADL, Activities of Daily Living; *IADL*, Instrumental Activities of Daily Living; *SPPB*, Short Physical Performance Battery; *n*, number of participants; *CI*, confidence intervals; *OR*, odds ratio.

[1] The quadratic time variable was subsequently excluded from models as there was no evidence of non-linearity.

[2] Age, biological sex, ethnicity, marital status, employment status, education, and wealth.

[3] Physical activity, body-mass index, cognitive function, and depressive symptoms.

Note: All values are weighted estimates.

Table S9. Mean changes in mobility impairments, ADL disabilities, and IADL disabilities, and odds ratios for falls per one-point increase in total SPPB score, using repeated measures outcomes from seven waves of follow-up (imputed samples).

	**Estimate (95% CI)**	***p* value**
Mobility impairments (*n*=4,932; 34,524 observations)		
Model 1: Univariable association	-0.54 (-0.56, -0.51)	<0.001
Model 2: Model 1 plus continuous linear time variable	-0.54 (-0.56, -0.52)	<0.001
Model 3: Model 2 plus continuous quadratic time variable	-0.54 (-0.56, -0.52)	<0.001
Model 4: Model 3 plus socio-demographic covariates [1]	-0.50 (-0.52, -0.47)	<0.001
Model 5: Model 4 plus health-related covariates [2]	-0.37 (-0.40, -0.34)	<0.001
Model 6: Model 5 plus mobility impairments at wave two	-0.15 (-0.18, -0.12)	<0.001
ADL disabilities (*n*=4,932; 34,524 observations)		
Model 1: Univariable association	-0.17 (-0.19, -0.14)	<0.001
Model 2: Model 1 plus continuous linear time variable	-0.19 (-0.21, -0.17)	<0.001
Model 3: Model 2 plus continuous quadratic time variable	-0.19 (-0.21, -0.17)	<0.001
Model 4: Model 3 plus socio-demographic covariates [1]	-0.20 (-0.22, -0.18)	<0.001
Model 5: Model 4 plus health-related covariates [2]	-0.15 (-0.17, -0.13)	<0.001
Model 6: Model 5 plus ADL disabilities at wave two	-0.08 (-0.10, -0.06)	<0.001
IADL disabilities (*n*=4,932; 34,524 observations)		
Model 1: Univariable association	-0.18 (-0.21, -0.15)	<0.001
Model 2: Model 1 plus continuous linear time variable	-0.22 (-0.24, -0.19)	<0.001
Model 3: Model 2 plus continuous quadratic time variable	-0.22 (-0.24, -0.19)	<0.001
Model 4: Model 3 plus socio-demographic covariates [1]	-0.21 (-0.24, -0.19)	<0.001
Model 5: Model 4 plus health-related covariates [2]	-0.15 (-0.17, -0.12)	<0.001
Model 6: Model 5 plus IADL disabilities at wave two	-0.07 (-0.09, -0.04)	<0.001
	**OR (95% CI)**	***p* value**
Falls (*n*=4,932; 34,524 observations)		
Model 1: Univariable association	0.85 (0.83, 0.87)	<0.001
Model 2: Model 1 plus continuous linear time variable	0.85 (0.83, 0.87)	<0.001
Model 3: Model 2 plus continuous quadratic time variable [3]	0.85 (0.83, 0.87)	<0.001
Model 4: Model 2 plus socio-demographic covariates [1]	0.88 (0.86, 0.91)	<0.001
Model 5: Model 4 plus health-related covariates [2]	0.91 (0.88, 0.93)	<0.001
Model 6: Model 5 plus falls at wave two	0.93 (0.90, 0.95)	<0.001

ADL, Activities of Daily Living; *IADL*, Instrumental Activities of Daily Living; *SPPB*, Short Physical Performance Battery; *n*, number of participants; *CI*, confidence intervals; *OR*, odds ratio.

[1] Age, biological sex, ethnicity, marital status, employment status, education, and wealth.

[2] Physical activity, body-mass index, cognitive function, and depressive symptoms.

[3] The quadratic time variable was subsequently excluded from models as there was no evidence of non-linearity.

Note: All values are weighted estimates. The "cmdok" option was used to force the "melogit" command to run on imputed data for multilevel logistic models with falls as the outcome.

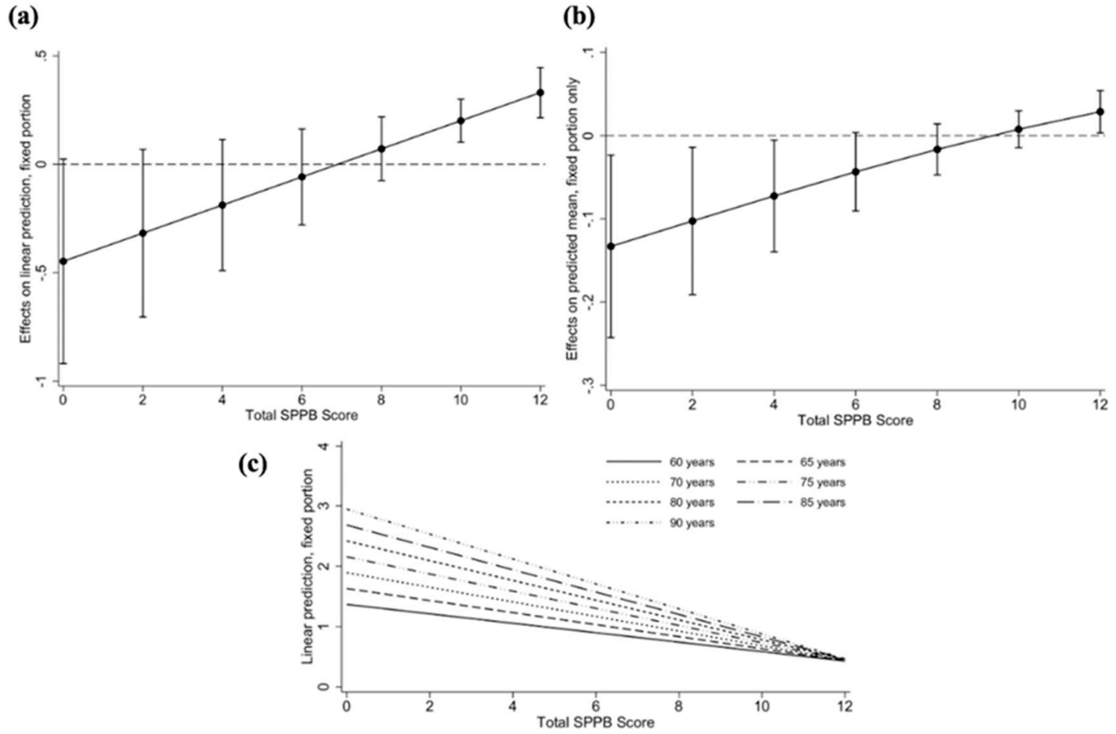

Figure S5. Marginal effects of biological sex on mobility impairments (**a**) and falls (**b**) at representative values of the baseline total SPPB score, and simple slopes for the relationship of baseline total SPPB score on IADL disabilities at different age values (**c**).

Note: Predicted margins were derived from Model 11 for mobility impairments (**a**), Model 8 for falls (**b**), and Model 11 for IADL disabilities (**c**). Values were computed using the postestimation "margins" command with the "vce(unconditional)" option. The random effects of the respective multilevel models were fixed to zero. The marginal effects depict the differences in mobility impairments (**a**) and falls (**b**) between males (reference) and females at various values of the baseline total SPPB score. Error bars indicate 95% confidence intervals. The simple slopes (**c**) show the amount of change in IADL disabilities for a one-point increase in baseline total SPPB score, while holding age constant at different values.

Table S10. Multilevel model results for baseline SPPB domain scores (balance, repeated chair stands, and gait) on mobility impairments, ADL disabilities, IADL disabilities, and falls over seven waves of follow-up: all exposures entered simultaneously.

	**Mobility impairments [1]**		**ADL disabilities [2]**		**IADL disabilities [2]**		**Falls [3]**	
	**Estimate (95% CI)**	***p* value**	**Estimate (95% CI)**	***p* value**	**Estimate (95% CI)**	***p* value**	**OR (95% CI)**	***p* value**
**Unadjusted**								
Balance								
0 (reference)	0.00		0.00		0.00		1.00	
1	0.34 (-0.37, 1.05)	0.347	-0.46 (-0.90, -0.02)	0.041	-0.06 (-0.52, 0.40)	0.809	1.38 (0.80, 2.36)	0.246
2	0.45 (-0.23, 1.13)	0.195	-0.62 (-1.05, -0.19)	0.005	-0.35 (-0.79, 0.08)	0.112	1.49 (0.88, 2.52)	0.136
3	0.29 (-0.40, 0.97)	0.413	-0.56 (-0.99, -0.14)	0.009	-0.35 (-0.77, 0.08)	0.109	1.16 (0.70, 1.93)	0.556
4	-0.28 (-0.94, 0.37)	0.398	-0.70 (-1.12, -0.29)	0.001	-0.56 (-0.97, -0.15)	0.007	0.77 (0.47, 1.26)	0.300
Repeated Chair Stands								
0 (reference)	0.00		0.00		0.00		1.00	
1	-1.03 (-1.39, -0.66)	<0.001	-0.40 (-0.57, -0.23)	<0.001	-0.23 (-0.43, -0.03)	0.026	0.70 (0.52, 0.94)	0.016
2	-1.48 (-1.83, -1.14)	<0.001	-0.52 (-0.68, -0.36)	<0.001	-0.27 (-0.46, -0.08)	0.005	0.73 (0.56, 0.97)	0.029
3	-1.81 (-2.14, -1.48)	<0.001	-0.60 (-0.75, -0.45)	<0.001	-0.33 (-0.51, -0.15)	<0.001	0.77 (0.59, 1.01)	0.059
4	-2.41 (-2.73, -2.09)	<0.001	-0.70 (-0.85, -0.55)	<0.001	-0.45 (-0.62, -0.27)	<0.001	0.64 (0.49, 0.83)	0.001
Gait								
0 (reference)	0.00		0.00		0.00		1.00	
1	-0.05 (-0.66, 0.56)	0.873	-0.25 (-0.57, 0.08)	0.143	0.36 (-0.07, 0.79)	0.097	0.73 (0.46, 1.15)	0.174
2	-0.47 (-1.03, 0.09)	0.103	-0.39 (-0.66, -0.11)	0.006	-0.11 (-0.48, 0.26)	0.559	0.69 (0.46, 1.04)	0.076
3	-1.56 (-2.11, -1.01)	<0.001	-0.68 (-0.93, -0.42)	<0.001	-0.50 (-0.86, -0.15)	0.005	0.51 (0.35, 0.75)	0.001
4	-2.55 (-3.08, -2.02)	<0.001	-0.88 (-1.12, -0.63)	<0.001	-0.84 (-1.19, -0.50)	<0.001	0.39 (0.27, 0.55)	<0.001
**Fully adjusted [4]**								
Balance								
0 (reference)	0.00		0.00		0.00		1.00	
1	0.31 (-0.29, 0.91)	0.312	-0.20 (-0.55, 0.14)	0.250	0.08 (-0.29, 0.45)	0.676	1.15 (0.68, 1.95)	0.597
2	0.46 (-0.10, 1.01)	0.107	-0.36 (-0.70, -0.03)	0.035	-0.17 (-0.50, 0.17)	0.331	1.23 (0.75, 2.03)	0.410
3	0.44 (-0.12, 1.00)	0.125	-0.27 (-0.60, 0.05)	0.101	-0.11 (-0.44, 0.23)	0.536	1.08 (0.66, 1.76)	0.760
4	0.14 (-0.40, 0.68)	0.608	-0.35 (-0.67, -0.03)	0.032	-0.17 (-0.50, 0.15)	0.294	0.86 (0.54, 1.38)	0.535
Repeated Chair Stands								
0 (reference)	0.00		0.00		0.00		1.00	

1	-0.32 (-0.59, -0.05)	0.022	-0.19 (-0.33, -0.05)	0.006	-0.13 (-0.29, 0.03)	0.102	0.69 (0.52, 0.90)	0.006
2	-0.45 (-0.71, -0.19)	0.001	-0.24 (-0.37, -0.10)	0.001	-0.12 (-0.27, 0.04)	0.137	0.83 (0.64, 1.08)	0.158
3	-0.44 (-0.70, -0.19)	0.001	-0.26 (-0.39, -0.13)	<0.001	-0.10 (-0.25, 0.05)	0.203	0.85 (0.66, 1.10)	0.210
4	-0.66 (-0.91, -0.41)	<0.001	-0.29 (-0.42, -0.17)	<0.001	-0.14 (-0.28, -0.00)	0.049	0.81 (0.63, 1.04)	0.105
Gait								
0 (reference)	0.00		0.00		0.00		1.00	
1	-0.35 (-0.80, 0.11)	0.135	-0.27 (-0.53, -0.02)	0.038	0.01 (-0.33, 0.34)	0.964	0.65 (0.42, 1.01)	0.057
2	-0.17 (-0.57, 0.23)	0.416	-0.20 (-0.43, 0.03)	0.089	-0.01 (-0.29, 0.27)	0.944	0.70 (0.48, 1.03)	0.070
3	-0.33 (-0.71, 0.05)	0.086	-0.26 (-0.47, -0.05)	0.016	-0.18 (-0.44, 0.09)	0.196	0.63 (0.44, 0.90)	0.011
4	-0.70 (-1.07, -0.33)	<0.001	-0.35 (-0.55, -0.14)	0.001	-0.33 (-0.59, -0.07)	0.012	0.52 (0.37, 0.74)	<0.001

ADL, Activities of Daily Living; *IADL*, Instrumental Activities of Daily Living; *SPPB*, Short Physical Performance Battery; *CI*, confidence intervals; *OR*, odds ratio.

[1] Number of participants: 3,548. Total observations: 16,934.

[2] Number of participants: 3,547. Total observations: 16,934.

[3] Number of participants: 3,505. Total observations: 16,332.

[4] Adjusted for linear time, quadratic time (excluded from the models with mobility impairments or falls as the outcome as there was no evidence of non-linearity), age, biological sex, ethnicity, marital status, employment status, education, wealth, physical activity, body-mass index, cognitive function, depressive symptoms, and the outcome from wave two.

Note: All values are weighted estimates.

Table S11. Multilevel logistic regression model results for baseline SPPB domain scores (balance, repeated chair stands, and gait) on mobility impairments, ADL disabilities, and IADL disabilities over seven waves of follow-up: each exposure entered in separate models.

	Mobility impairments [1]		ADL disabilities [2]		IADL disabilities [2]	
	OR (95% CI)	*p* value	OR (95% CI)	*p* value	OR (95% CI)	*p* value
**Unadjusted**						
Balance						
0 (reference)	1.00		1.00		1.00	
1	1.29 (0.43, 3.85)	0.645	0.66 (0.28, 1.56)	0.345	0.62 (0.27, 1.46)	0.275
2	0.75 (0.28, 2.02)	0.576	0.24 (0.10, 0.55)	0.001	0.29 (0.13, 0.67)	0.004
3	0.27 (0.11, 0.69)	0.006	0.12 (0.05, 0.26)	<0.001	0.16 (0.07, 0.35)	<0.001
4	0.05 (0.02, 0.11)	<0.001	0.03 (0.01, 0.06)	<0.001	0.03 (0.01, 0.06)	<0.001
Repeated Chair Stands						
0 (reference)	1.00		1.00		1.00	
1	0.20 (0.12, 0.35)	<0.001	0.19 (0.13, 0.28)	<0.001	0.21 (0.15, 0.31)	<0.001
2	0.09 (0.05, 0.15)	<0.001	0.09 (0.06, 0.13)	<0.001	0.11 (0.08, 0.16)	<0.001
3	0.04 (0.03, 0.07)	<0.001	0.05 (0.04, 0.07)	<0.001	0.06 (0.04, 0.09)	<0.001
4	0.01 (0.01, 0.02)	<0.001	0.02 (0.02, 0.03)	<0.001	0.02 (0.02, 0.03)	<0.001
Gait						
0 (reference)	1.00		1.00		1.00	
1	3.24 (0.88, 11.88)	0.076	0.79 (0.40, 1.56)	0.496	1.76 (0.86, 3.62)	0.123
2	0.92 (0.39, 2.16)	0.844	0.41 (0.23, 0.74)	0.003	0.63 (0.34, 1.15)	0.133
3	0.20 (0.09, 0.42)	<0.001	0.11 (0.07, 0.19)	<0.001	0.19 (0.11, 0.34)	<0.001
4	0.04 (0.02, 0.07)	<0.001	0.03 (0.02, 0.04)	<0.001	0.03 (0.02, 0.05)	<0.001
**Fully adjusted [3]**						
Balance						
0 (reference)	1.00		1.00		1.00	
1	1.29 (0.46, 3.58)	0.629	0.90 (0.43, 1.85)	0.767	0.83 (0.37, 1.88)	0.659
2	1.12 (0.44, 2.85)	0.809	0.48 (0.24, 0.97)	0.040	0.54 (0.25, 1.19)	0.128
3	0.71 (0.30, 1.71)	0.448	0.43 (0.22, 0.85)	0.015	0.49 (0.23, 1.06)	0.068
4	0.47 (0.21, 1.07)	0.072	0.33 (0.18, 0.62)	0.001	0.30 (0.15, 0.63)	0.001
Repeated Chair Stands						
0 (reference)	1.00		1.00		1.00	

1	0.45 (0.27, 0.75)	0.002	0.44 (0.31, 0.62)	<0.001	0.47 (0.32, 0.67)	<0.001
2	0.35 (0.22, 0.55)	<0.001	0.37 (0.26, 0.52)	<0.001	0.44 (0.31, 0.62)	<0.001
3	0.30 (0.19, 0.48)	<0.001	0.32 (0.23, 0.45)	<0.001	0.37 (0.27, 0.51)	<0.001
4	0.22 (0.14, 0.35)	<0.001	0.23 (0.16, 0.32)	<0.001	0.28 (0.20, 0.38)	<0.001
Gait						
0 (reference)	1.00		1.00		1.00	
1	0.85 (0.29, 2.48)	0.767	0.34 (0.18, 0.63)	0.001	0.68 (0.35, 1.34)	0.271
2	0.53 (0.25, 1.12)	0.095	0.40 (0.24, 0.67)	<0.001	0.59 (0.35, 1.02)	0.060
3	0.39 (0.20, 0.76)	0.006	0.30 (0.19, 0.49)	<0.001	0.44 (0.27, 0.72)	0.001
4	0.23 (0.12, 0.45)	<0.001	0.19 (0.12, 0.31)	<0.001	0.23 (0.14, 0.38)	<0.001

ADL, Activities of Daily Living; *IADL*, Instrumental Activities of Daily Living; *SPPB*, Short Physical Performance Battery; *CI*, confidence intervals; *OR*, odds ratio.

[1] Number of participants: 3,548. Total observations: 16,934.

[2] Number of participants: 3,547. Total observations: 16,934.

[3] Adjusted for linear time (quadratic time was excluded from the models as there was no evidence of non-linearity), age, biological sex, ethnicity, marital status, employment status, education, wealth, physical activity, body-mass index, cognitive function, depressive symptoms, and the outcome from wave two.

Note: All values are weighted estimates.

Table S12. Multilevel logistic regression model results for baseline SPPB domain scores (balance, repeated chair stands, and gait) on mobility impairments, ADL disabilities, and IADL disabilities over seven waves of follow-up: all exposures entered simultaneously.

	**Mobility impairments [1]**		**ADL disabilities [2]**		**IADL disabilities [2]**	
	**OR (95% CI)**	***p* value**	**OR (95% CI)**	***p* value**	**OR (95% CI)**	***p* value**
**Unadjusted**						
Balance						
0 (reference)	1.00		1.00		1.00	
1	4.53 (1.44, 14.24)	0.010	1.68 (0.76, 3.68)	0.198	1.39 (0.63, 3.07)	0.414
2	4.50 (1.57, 12.91)	0.005	0.98 (0.46, 2.11)	0.958	0.97 (0.44, 2.13)	0.939
3	2.72 (0.98, 7.52)	0.054	0.94 (0.44, 2.00)	0.863	0.94 (0.44, 2.02)	0.874
4	1.12 (0.43, 2.91)	0.810	0.53 (0.26, 1.08)	0.082	0.37 (0.18, 0.76)	0.007
Repeated Chair Stands						
0 (reference)	1.00		1.00		1.00	
1	0.47 (0.26, 0.87)	0.016	0.47 (0.32, 0.70)	<0.001	0.61 (0.41, 0.89)	0.011
2	0.28 (0.16, 0.50)	<0.001	0.30 (0.20, 0.44)	<0.001	0.47 (0.32, 0.67)	<0.001
3	0.17 (0.10, 0.29)	<0.001	0.22 (0.15, 0.32)	<0.001	0.34 (0.24, 0.49)	<0.001
4	0.06 (0.04, 0.11)	<0.001	0.11 (0.07, 0.16)	<0.001	0.17 (0.12, 0.24)	<0.001
Gait						
0 (reference)	1.00		1.00		1.00	
1	1.81 (0.54, 6.07)	0.334	0.60 (0.32, 1.14)	0.118	1.34 (0.68, 2.64)	0.402
2	0.88 (0.38, 2.04)	0.773	0.52 (0.31, 0.89)	0.016	0.75 (0.43, 1.32)	0.317
3	0.35 (0.16, 0.75)	0.007	0.26 (0.16, 0.43)	<0.001	0.40 (0.24, 0.68)	0.001
4	0.13 (0.06, 0.27)	<0.001	0.11 (0.07, 0.18)	<0.001	0.12 (0.07, 0.20)	<0.001
**Fully adjusted [3]**						
Balance						
0 (reference)	1.00		1.00		1.00	
1	2.27 (0.77, 6.71)	0.139	1.46 (0.71, 3.02)	0.302	1.17 (0.53, 2.58)	0.692
2	2.42 (0.91, 6.46)	0.078	0.91 (0.45, 1.87)	0.804	0.86 (0.39, 1.87)	0.705
3	1.78 (0.69, 4.58)	0.231	1.01 (0.51, 2.02)	0.974	0.91 (0.42, 1.97)	0.812
4	1.32 (0.54, 3.26)	0.540	0.86 (0.45, 1.66)	0.655	0.64 (0.30, 1.33)	0.229
Repeated Chair Stands						
0 (reference)	1.00		1.00		1.00	

1	0.58 (0.34, 1.00)	0.049	0.55 (0.38, 0.80)	0.002	0.64 (0.44, 0.93)	0.021
2	0.48 (0.29, 0.80)	0.005	0.48 (0.34, 0.70)	<0.001	0.65 (0.45, 0.94)	0.023
3	0.43 (0.26, 0.71)	0.001	0.44 (0.31, 0.63)	<0.001	0.58 (0.40, 0.83)	0.003
4	0.32 (0.20, 0.52)	<0.001	0.31 (0.22, 0.44)	<0.001	0.43 (0.30, 0.62)	<0.001
Gait						
0 (reference)	1.00		1.00		1.00	
1	0.76 (0.26, 2.19)	0.612	0.34 (0.18, 0.63)	0.001	0.70 (0.36, 1.36)	0.287
2	0.53 (0.25, 1.12)	0.098	0.45 (0.27, 0.77)	0.003	0.67 (0.38, 1.16)	0.153
3	0.46 (0.23, 0.90)	0.024	0.41 (0.25, 0.68)	<0.001	0.57 (0.34, 0.95)	0.033
4	0.31 (0.16, 0.60)	0.001	0.30 (0.18, 0.48)	<0.001	0.34 (0.20, 0.56)	<0.001

ADL, Activities of Daily Living; *IADL*, Instrumental Activities of Daily Living; *SPPB*, Short Physical Performance Battery; *CI*, confidence intervals; *OR*, odds ratio.

[1] Number of participants: 3,548. Total observations: 16,934.

[2] Number of participants: 3,547. Total observations: 16,934.

[3] Adjusted for linear time (quadratic time was excluded from the models as there was no evidence of non-linearity), age, biological sex, ethnicity, marital status, employment status, education, wealth, physical activity, body-mass index, cognitive function, depressive symptoms, and the outcome from wave two.

Note: All values are weighted estimates.

Table S13. Multilevel model results for baseline SPPB domain scores (balance, repeated chair stands, and gait) on mobility impairments, ADL disabilities, IADL disabilities, and falls over seven waves of follow-up: each exposure entered in separate models (imputed samples).

	**Mobility impairments** [1]		**ADL disabilities** [1]		**IADL disabilities** [1]		**Falls** [1]	
	**Estimate (95% CI)**	***p* value**	**Estimate (95% CI)**	***p* value**	**Estimate (95% CI)**	***p* value**	**OR (95% CI)**	***p* value**
**Unadjusted**								
Balance								
0 (reference)	0.00		0.00		0.00		1.00	
1	-1.72 (-2.30, -1.14)	<0.001	-1.27 (-1.68, -0.85)	<0.001	-1.26 (-1.79, -0.73)	<0.001	0.74 (0.52, 1.07)	0.105
2	-2.32 (-2.87, -1.76)	<0.001	-1.68 (-2.07, -1.29)	<0.001	-1.80 (-2.31, -1.29)	<0.001	0.62 (0.43, 0.89)	0.011
3	-3.25 (-3.76, -2.74)	<0.001	-1.78 (-2.15, -1.42)	<0.001	-1.91 (-2.37, -1.46)	<0.001	0.47 (0.34, 0.66)	<0.001
4	-4.54 (-4.96, -4.11)	<0.001	-1.97 (-2.32, -1.62)	<0.001	-2.13 (-2.55, -1.70)	<0.001	0.24 (0.17, 0.33)	<0.001
Repeated Chair Stands								
0 (reference)	0.00		0.00		0.00		1.00	
1	-2.29 (-2.63, -1.95)	<0.001	-1.04 (-1.25, -0.83)	<0.001	-1.17 (-1.45, -0.89)	<0.001	0.61 (0.50, 0.75)	<0.001
2	-3.16 (-3.47, -2.85)	<0.001	-1.16 (-1.36, -0.95)	<0.001	-1.27 (-1.53, -1.00)	<0.001	0.45 (0.37, 0.56)	<0.001
3	-3.67 (-3.96, -3.39)	<0.001	-1.24 (-1.44, -1.04)	<0.001	-1.29 (-1.54, -1.04)	<0.001	0.39 (0.32, 0.48)	<0.001
4	-4.38 (-4.64, -4.12)	<0.001	-1.30 (-1.51, -1.09)	<0.001	-1.35 (-1.60, -1.10)	<0.001	0.30 (0.24, 0.36)	<0.001
Gait								
0 (reference)	0.00		0.00		0.00		1.00	
1	-0.08 (-0.63, 0.47)	0.780	-0.50 (-0.91, -0.09)	0.016	-0.22 (-0.75, 0.30)	0.403	0.91 (0.68, 1.21)	0.511
2	-1.42 (-1.92, -0.91)	<0.001	-1.25 (-1.58, -0.92)	<0.001	-1.39 (-1.83, -0.96)	<0.001	0.73 (0.55, 0.96)	0.024
3	-3.06 (-3.53, -2.59)	<0.001	-1.53 (-1.84, -1.22)	<0.001	-1.72 (-2.12, -1.31)	<0.001	0.45 (0.34, 0.59)	<0.001
4	-4.52 (-4.96, -4.08)	<0.001	-1.76 (-2.07, -1.45)	<0.001	-1.99 (-2.38, -1.60)	<0.001	0.27 (0.21, 0.35)	<0.001
**Fully adjusted** [2]								
Balance			No convergence					
0 (reference)	0.00				0.00		1.00	
1	-0.33 (-0.69, 0.02)	0.066			-0.42 (-0.77, -0.07)	0.019	0.80 (0.56, 1.14)	0.217
2	-0.49 (-0.80, -0.18)	0.002			-0.75 (-1.10, -0.40)	<0.001	0.77 (0.54, 1.10)	0.143
3	-0.57 (-0.88, -0.26)	<0.001			-0.70 (-1.02, -0.37)	<0.001	0.70 (0.50, 0.98)	0.037
4	-0.94 (-1.22, -0.65)	<0.001			-0.75 (-1.05, -0.45)	<0.001	0.53 (0.37, 0.75)	0.001
Repeated Chair Stands								
0 (reference)	0.00		0.00		0.00		1.00	

1	-0.47 (-0.70, -0.24)	<0.001	-0.40 (-0.56, -0.24)	<0.001	-0.36 (-0.56, -0.16)	0.001	0.77 (0.63, 0.93)	0.008
2	-0.66 (-0.89, -0.44)	<0.001	-0.43 (-0.57, -0.28)	<0.001	-0.36 (-0.55, -0.17)	<0.001	0.77 (0.63, 0.94)	0.011
3	-0.75 (-0.97, -0.53)	<0.001	-0.44 (-0.58, -0.30)	<0.001	-0.31 (-0.49, -0.13)	0.001	0.73 (0.60, 0.87)	0.001
4	-0.95 (-1.16, -0.73)	<0.001	-0.45 (-0.59, -0.30)	<0.001	-0.32 (-0.50, -0.14)	<0.001	0.68 (0.55, 0.83)	<0.001
Gait			No convergence					
0 (reference)	0.00				0.00		1.00	
1	-0.09 (-0.43, 0.25)	0.600			-0.07 (-0.42, 0.28)	0.685	0.79 (0.60, 1.05)	0.109
2	-0.21 (-0.52, 0.10)	0.175			-0.49 (-0.80, -0.18)	0.002	0.83 (0.63, 1.08)	0.163
3	-0.49 (-0.79, -0.19)	0.002			-0.55 (-0.83, -0.26)	<0.001	0.69 (0.53, 0.89)	0.005
4	-0.92 (-1.22, -0.62)	<0.001			-0.66 (-0.94, -0.38)	<0.001	0.56 (0.43, 0.73)	<0.001

ADL, Activities of Daily Living; *IADL*, Instrumental Activities of Daily Living; *SPPB*, Short Physical Performance Battery; *CI*, confidence intervals; *OR*, odds ratio.

[1] Number of participants: 4,932. Total observations: 34,524. Imputations: 25.

[2] Adjusted for linear time, quadratic time (excluded from the models with falls as the outcome as there was no evidence of non-linearity), age, biological sex, ethnicity, marital status, employment status, education, wealth, physical activity, body-mass index, cognitive function, depressive symptoms, and the outcome from wave two.

Note: All values are weighted estimates. The "cmdok" option was used to force the "melogit" command to run on imputed data for multilevel logistic models with falls as the outcome.

Table S14. Multilevel model results for baseline SPPB domain scores (balance, repeated chair stands, and gait) on mobility impairments, ADL disabilities, IADL disabilities, and falls over seven waves of follow-up: all exposures entered simultaneously (imputed samples).

	**Mobility impairments [1]**		**ADL disabilities [1]**		**IADL disabilities [1]**		**Falls [1]**	
	**Estimate (95% CI)**	***p* value**	**Estimate (95% CI)**	***p* value**	**Estimate (95% CI)**	***p* value**	**OR (95% CI)**	***p* value**
**Unadjusted**								
Balance								
0 (reference)	0.00		0.00		0.00		1.00	
1	-0.49 (-1.01, 0.03)	0.065	-0.79 (-1.17, -0.40)	<0.001	-0.76 (-1.26, -0.25)	0.003	0.90 (0.63, 1.29)	0.556
2	-0.50 (-0.99, -0.00)	0.049	-0.99 (-1.36, -0.63)	<0.001	-1.06 (-1.55, -0.58)	<0.001	0.82 (0.56, 1.19)	0.285
3	-0.60 (-1.11, -0.10)	0.020	-0.90 (-1.26, -0.53)	<0.001	-0.92 (-1.39, -0.45)	<0.001	0.74 (0.51, 1.05)	0.094
4	-0.98 (-1.45, -0.51)	<0.001	-0.90 (-1.25, -0.56)	<0.001	-0.92 (-1.36, -0.47)	<0.001	0.47 (0.32, 0.68)	<0.001
Repeated Chair Stands								
0 (reference)	0.00		0.00		0.00		1.00	
1	-1.04 (-1.39, -0.70)	<0.001	-0.47 (-0.67, -0.27)	<0.001	-0.44 (-0.71, -0.16)	0.002	1.01 (0.82, 1.25)	0.891
2	-1.50 (-1.84, -1.16)	<0.001	-0.53 (-0.71, -0.34)	<0.001	-0.41 (-0.69, -0.14)	0.003	0.89 (0.72, 1.10)	0.281
3	-1.79 (-2.12, -1.47)	<0.001	-0.57 (-0.75, -0.39)	<0.001	-0.38 (-0.63, -0.13)	0.003	0.85 (0.70, 1.05)	0.131
4	-2.34 (-2.66, -2.02)	<0.001	-0.63 (-0.82, -0.44)	<0.001	-0.41 (-0.66, -0.16)	0.001	0.69 (0.56, 0.86)	0.001
Gait								
0 (reference)	0.00		0.00		0.00		1.00	
1	-0.14 (-0.62, 0.35)	0.583	-0.39 (-0.76, -0.03)	0.035	-0.10 (-0.61, 0.40)	0.686	0.89 (0.66, 1.19)	0.415
2	-0.81 (-1.27, -0.35)	0.001	-0.87 (-1.16, -0.57)	<0.001	-1.03 (-1.46, -0.60)	<0.001	0.87 (0.66, 1.15)	0.331
3	-1.84 (-2.30, -1.39)	<0.001	-0.98 (-1.25, -0.71)	<0.001	-1.22 (-1.62, -0.81)	<0.001	0.66 (0.50, 0.87)	0.004
4	-2.77 (-3.22, -2.33)	<0.001	-1.11 (-1.38, -0.84)	<0.001	-1.43 (-1.83, -1.04)	<0.001	0.49 (0.37, 0.65)	<0.001
**Fully adjusted [2]**			No convergence					
Balance								
0 (reference)	0.00				0.00		1.00	
1	-0.18 (-0.55, 0.19)	0.344			-0.33 (-0.69, 0.03)	0.071	0.86 (0.61, 1.23)	0.409
2	-0.28 (-0.60, 0.05)	0.097			-0.61 (-0.97, -0.26)	0.001	0.84 (0.58, 1.21)	0.337
3	-0.25 (-0.59, 0.10)	0.159			-0.51 (-0.86, -0.17)	0.003	0.80 (0.56, 1.14)	0.212
4	-0.52 (-0.84, -0.20)	0.001			-0.54 (-0.86, -0.22)	0.001	0.62 (0.43, 0.91)	0.015
Repeated Chair Stands								
0 (reference)	0.00				0.00		1.00	
1	-0.23 (-0.47, 0.01)	0.065			-0.13 (-0.33, 0.08)	0.230	0.95 (0.78, 1.15)	0.568

2	-0.38 (-0.62, -0.14)	0.002			-0.11 (-0.31, 0.09)	0.263	0.98 (0.80, 1.20)	0.851
3	-0.44 (-0.67, -0.20)	<0.001			-0.05 (-0.25, 0.14)	0.578	0.95 (0.78, 1.15)	0.566
4	-0.64 (-0.88, -0.40)	<0.001			-0.08 (-0.27, 0.11)	0.405	0.88 (0.72, 1.08)	0.228
Gait								
0 (reference)	0.00				0.00		1.00	
1	-0.07 (-0.41, 0.26)	0.664			-0.01 (-0.36, 0.34)	0.957	0.82 (0.61, 1.09)	0.173
2	-0.09 (-0.40, 0.22)	0.589			-0.35 (-0.66, -0.05)	0.025	0.91 (0.70, 1.19)	0.488
3	-0.27 (-0.58, 0.03)	0.073			-0.38 (-0.66, -0.10)	0.007	0.80 (0.61, 1.04)	0.088
4	-0.64 (-0.93, -0.34)	<0.001			-0.49 (-0.77, -0.22)	<0.001	0.68 (0.51, 0.89)	0.007

ADL, Activities of Daily Living; *IADL*, Instrumental Activities of Daily Living; *SPPB*, Short Physical Performance Battery; *CI*, confidence intervals; *OR*, odds ratio.

[1] Number of participants: 4,932. Total observations: 34,524. Imputations: 25.

[2] Adjusted for linear time, quadratic time (excluded from the model with falls as the outcome as there was no evidence of non-linearity), age, biological sex, ethnicity, marital status, employment status, education, wealth, physical activity, body-mass index, cognitive function, depressive symptoms, and the outcome from wave two.

Note: All values are weighted estimates. The "cmdok" option was used to force the "melogit" command to run on imputed data for multilevel logistic models with falls as the outcome.
